# Human papillomavirus (HPV) testing for cervical cancer screening in a middle-income country: comment on a large real-world implementation study in China

**DOI:** 10.1186/s12916-021-02051-z

**Published:** 2021-07-15

**Authors:** Louise T. Thomsen, Susanne K. Kjær

**Affiliations:** 1 Unit of Virus, Lifestyle and Genes, Danish Cancer Society Research Center, Strandboulevarden 49, 2100 Copenhagen, Denmark; 2 Department of Gynecology, Rigshospitalet, Copenhagen University Hospital, Blegdamsvej 3, 2100 Copenhagen, Denmark

**Keywords:** Human papillomavirus, HPV, Cervical cancer, Cancer cervix uteri, Screening, Prevention, Middle income

## Background

Cervical cancer can effectively be prevented by human papillomavirus (HPV) vaccination and early detection and treatment of precancerous lesions (screening). Nevertheless, cervical cancer remains a global public health problem and an important threat to women’s health worldwide. It is the fourth most common cancer in women with more than 600,000 cases and 342,000 deaths in 2020 [1]. Approximately 90% of cervical cancer deaths occur in low- and middle-income countries, underlining the substantial global inequality in disease burden [1]. In May 2018, the World Health Organization (WHO) called for global action to eliminate cervical cancer as a public health problem [2]. The WHO urged member states to scale up efforts to implement preventive strategies against cervical cancer, including HPV vaccination, screening, and treatment for precancerous lesions and cancer [2].

## Cervical cancer screening by HPV testing

Cervical cancer screening has traditionally been based on detection of cytological abnormalities in cervical cell samples, so-called cytology-based screening. This method has effectively reduced cervical cancer incidence in high-income countries when implemented in organized programs [3, 4]. However, in low- and middle-income countries, implementing cytology-based screening has been challenging, because it requires substantial provider training, continued quality assurance, and repeated testing at relatively short intervals [3, 4]. Visual inspection with acetic acid is used as a screening method in some low-resource settings, but this method has substantial inter-observer variability and limited sensitivity [3].

During the past two decades, HPV testing has emerged as a new and highly effective screening method against cervical cancer. Randomized trials in high-income [5] and middle-income [6] countries have demonstrated that HPV testing is more sensitive and prevents more cervical cancers than cytology. These findings have been corroborated by real-world implementation studies in high-income countries, e.g., the Netherlands [7] and Denmark [8]. A challenge with HPV testing, however, is that most HPV infections are transient, and therefore, triage testing of HPV positive women is recommended to prevent over-referral and over-treatment [4, 7, 8].

## A large observational study of HPV testing in a middle-income country

Until now, there has been limited evidence on the real-world performance of HPV testing for cervical cancer screening in middle-income countries [9]. In this issue of BMC Medicine, Zhao et al. report results from a large implementation study of HPV-based cervical cancer screening in China [10]. This population-based observational study included approximately 1.1 million women, of whom 800,000 received HPV-based and 300,000 received cytology-based screening. In the HPV group, HPV-positive women were triaged by cytology alone or HPV16/18 genotyping and cytology.

To our knowledge, the study by Zhao et al. is the largest to date on the performance of HPV testing versus cytology for cervical cancer screening in a middle-income country. In line with findings from high-income settings [5, 7, 8], the authors found that HPV testing detected more cases of cervical intraepithelial neoplasia grade 2 or worse (CIN2+) than cytology-based screening, underlining the superior sensitivity of HPV testing. However, in contrast to results in high-income settings [7, 8], the referral rate to immediate colposcopy was lower for HPV than cytology-based screening. As noted by the authors [10], this likely reflects that in the Chinese cytology-based screening program, all women with atypical squamous cells of undetermined significance or worse were referred directly to colposcopy, because not all laboratories could perform HPV triage testing for mild abnormalities, and compliance with repeat cytology could not be ensured [10]. Thus, in the Chinese setting, HPV-based screening was more efficient than cytology-based screening, since it simultaneously increased CIN2+-detection, decreased immediate colposcopy referrals, and markedly improved the positive predictive value (PPV) of referral.

Interestingly, Zhao et al. [10] also performed an analysis stratified by county income level (lower-middle or upper-middle income). They found that the increased CIN2+-detection for HPV compared with cytology-based screening was most pronounced in lower-middle income areas, likely reflecting the lower quality of cytology in these settings. However, the PPV of colposcopy referral increased with HPV-based screening in both lower-middle income and upper-middle income areas, supporting the higher efficiency of HPV-based screening irrespective of income level [10].

## Clinical implications

The findings of Zhao et al. provide strong support for implementing HPV-based screening in China and other middle-income countries. In addition to its excellent sensitivity, an advantage of HPV testing is the high and long-lasting negative predictive value, permitting an extension of screening intervals to 5 years or more [5]. Even one or two HPV screens in a lifetime may confer substantial preventive benefit [2, 4]. Furthermore, HPV-based screening can successfully be implemented as a “see-and-treat” approach, where screening, triage, and treatment, e.g., by cryotherapy, are provided at the same visit [2–4]. Point-of-care HPV testing platforms are available which provide rapid results and require limited skills of laboratory technicians [3]. In addition, HPV testing can be performed on self-collected specimens, thereby obliterating the need for a gynecological exam at the initial screen [2]. All of these features represent substantial benefits in remote areas, such as rural China, where women may need to travel long distances for screening and treatment, and where health care provider resources are limited.

Although the advantages of HPV testing are well-documented, practical implementation of an HPV-based screening program can pose substantial challenges. Policy-makers planning to implement HPV-based screening face multiple choices regarding program design, including choice of HPV test, triage method, follow-up and referral recommendation, target age range, screening interval, communication strategy, and training strategy for health care providers [9]. The optimal choices in a specific setting will depend on the national and local context, including the availability of financial resources, health care staff, and technical capacity and infrastructure [9, 10]. Regardless of the chosen strategy, it is essential to ensure adequate treatment of screen-identified precancerous lesions and cancers, since screening without access to treatment is unethical [2].

## Conclusion

The study by Zhao et al. provides further evidence for the benefits of HPV-based cervical cancer screening in middle-income settings. Introducing HPV-based screening in countries around the world is a crucial step to achieve the global goal of cervical cancer elimination, potentially saving the lives of thousands of women worldwide.

## Data Availability

Not applicable.
